# Single crystals of SnTe_3_O_8_ in the millimetre range grown by chemical vapor transport reactions

**DOI:** 10.1107/S2056989021011828

**Published:** 2021-11-12

**Authors:** Michael Ketter, Matthias Weil

**Affiliations:** a Institute for Chemical Technologies and Analytics, Division of Structural Chemistry, TU Wien, Getreidemarkt 9/164-SC, A-1060 Vienna, Austria

**Keywords:** crystal structure, oxidotellurates, isotypism, electron lone pair

## Abstract

Tin(IV) trioxidotellurate(IV), SnTe_3_O_8_, is a member of the isotypic *M* ^IV^Te^IV^_3_O_8_ (*M* = Ti, Zr, Hf, Sn) series crystallizing with eight formula units per unit cell in space group *Ia*  . In comparison with the previous crystal structure model of SnTe_3_O_8_ based on powder X-ray diffraction data [Meunier & Galy (1971 ▸). *Acta Cryst.* B**27**, 602–608], the current model based on single-crystal X-ray data is improved in terms of precision and accuracy. Nearly regular [SnO_6_] octa­hedra (Sn site symmetry .  .) are situated in the voids of an oxidotellurate(IV) framework built up by corner-sharing [TeO_4_] bis­phenoids (Te site symmetry 2..). A qu­anti­tative structural comparison revealed a very high degree of similarity for the structures with *M* = Ti, Zr, Sn in the *M* ^IV^Te_3_O_8_ series.

## Abstract 2

SnTe_3_O_8_ crystallizes isotypically with other members of the series *M* ^IV^Te^IV^_3_O_8_ (*M* = Ti, Zr, Hf). It comprises [SnO_6_] octa­hedra and [TeO_4_] bis­phenoids as the principal structural building blocks.

## Chemical context

The crystal chemistry of oxidotellurates(IV) is dominated by the presence of the 5*s* ^2^ electron lone pair that, in the majority of cases, is stereochemically active, thus enabling one-sided coordination spheres around the Te^IV^ atom (Christy *et al.*, 2016 ▸). This peculiar building block often results in compounds with non-centrosymmetric structures or structures with polar directions exhibiting inter­esting physical properties (Ra *et al.*, 2003 ▸; Kim *et al.*, 2014 ▸). In this context, the microwave dielectric properties of *M* ^IV^Te_3_O_8_ (*M* = Sn, Zr) ceramics were investigated some time ago (Subodh & Sebastian, 2008 ▸).

The crystal structure of the isotypic series *M* ^IV^Te_3_O_8_ was originally determined for *M* = Ti from a single crystal in space group *Ia* 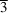 using photographic Weissenberg X-ray data, whereas for *M* = Sn, Zr and Hf, the crystal structures were refined from powder X-ray data (Meunier & Galy, 1971 ▸). In subsequent studies, crystal-structure refinements on the basis of single-crystal X-ray data were reported for the mineral winstanleyite with composition (Ti_0.96_Fe_0.04_)Te_3_O_8_ (Bindi & Cipriani, 2003 ▸), and for the synthetic compound ZrTe_3_O_8_ (Noguera *et al.*, 2003 ▸; Lu *et al.*, 2019 ▸). A powder X-ray study of the solid solution Sn_0.59_Ti_0.41_Te_3_O_8_ crystallizing in the *M* ^IV^Te_3_O_8_ structure type has also been reported (Ben Aribia *et al.*, 2008 ▸).

Single-crystal growth of oxidotellurates(IV) can be accomplished through various crystallization methods including, for example, experiments under hydro­thermal conditions (Weil *et al.*, 2018 ▸), cooling from the melt (Stöger *et al.*, 2009 ▸), from salt melts as fluxing agents (Weil, 2019 ▸), or from chemical vapor transport reactions (Missen *et al.*, 2020 ▸). The latter method (Binnewies *et al.*, 2012 ▸) is particularly suitable for growing large crystals of high quality and was the method of choice for crystal growth of SnTe_3_O_8_ for which a more precise and accurate structure refinement appeared to be desirable.

## Structural commentary

**Figure 1:**
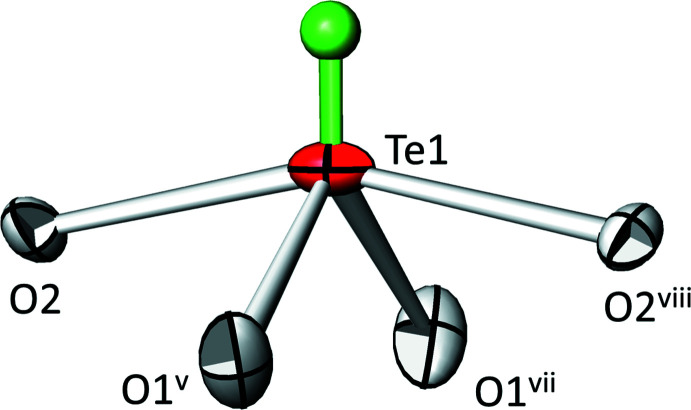
The coordination environment around Te1. Displacement ellipsoids are drawn at the 90% probability level; the electron lone pair is given as a green sphere of arbitrary radius. [Symmetry codes: (v) –*z* + 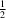 , *x*–1/2, *y*; (vii) −*z* + 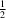 , −*x* + 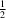 , −*y* + 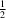 ; (viii) *x*, −*y*, −*z* + 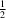 .]

The asymmetric unit of SnTe_3_O_8_ comprises one Sn^IV^ atom, one Te^IV^ atom, and two oxide anions, residing on sites 8*a* (site symmetry . 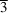 .), 24*d* (2..), 48*e* (1) and 16*c* (.3.), respectively. The tin atom is in an almost regular octa­hedral coordination by oxygen, with six equal Sn1—O1 distances, all *trans* angles equal to 180°, and *cis* angles ranging from 86.09 (4) to 93.91 (4)°. The Te1 site is coordinated by four O atoms in pairs of shorter (O1) and longer distances (O2) (Table 1 ▸). The resulting [TeO_4_] coordination polyhedron is a distorted bis­phenoid. Considering the 5*s* ^2^ electron lone pair at the Te^IV^ atom, the corresponding [ΨTeO_4_] polyhedron has a shape inter­mediate between a square pyramid and a trigonal bipyra­mid with the non-bonding electron pair occupying an equatorial position (Fig. 1 ▸). The geometry index *τ*_5_ of the [ΨTeO_4_] polyhedron is 0.471 (*τ*_5_ = 0 for an ideal square pyramid and *τ*_5_ = 1 for an ideal trigonal bipyramid; Addison *et al.*, 1984 ▸). The position of the electron lone pair was calculated with the *LPLoc* software (Hamani *et al.*, 2020 ▸), with resulting fractional coordinates of *x* = 0.28655, *y* = 0, *z* = 1/4. The radius of the electron lone pair was calculated to be 1.07 Å with a distance of 0.90 Å from the Te1 position. The coordination numbers of the oxide anions are two and three: O1 coord­inates to Sn1 and Te1 at the shorter of the two Te1—O distances whereas O2 coordinates to three Te1 atoms at the longer of the two Te1—O distances.

**Figure 2:**
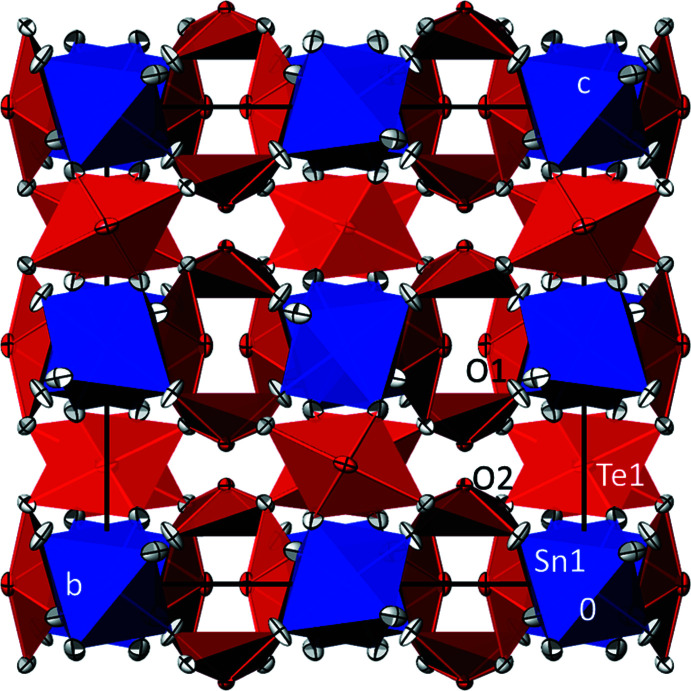
The crystal structure of SnTe_3_O_8_ in polyhedral representation, showing a projection along [ 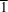 00]. Displacement ellipsoids are as in Fig. 1 ▸; [TeO_4_] polyhedra are red, [SnO_6_] octa­hedra are blue.

In the crystal structure of SnTe_3_O_8_, the [SnO_6_] octa­hedra are isolated from each other and arranged in rows running parallel to [100]. Each of the [TeO_4_] bis­phenoids shares corners (O2) with other [TeO_4_] bis­phenoids to form a three-dimensional oxidotellurate(IV) framework. The [SnO_6_] octa­hedra are situated in the voids of this framework, thereby sharing each of the six corners with an individual [TeO_4_] bis­phenoid. The crystal structure of SnTe_3_O_8_ is depicted in Fig. 2 ▸.

The unit-cell parameter *a* from the previous powder X-ray study, 11.144 (3) Å, as well as inter­atomic distances of Sn1—O1 = 2.032 Å (6×), Te1—O1 = 1.850 Å (2×), Te1—O2 = 2.124 Å (2×), and angles O1—Te1—O1′ = 102.9°, and O2—Te1—O2′ = 156.8° (Meunier & Galy, 1971 ▸) agree with the present single-crystal study (Table 1 ▸), but with lower precision and accuracy. In comparison with the previous model based on powder X-ray data, the values of the bond-valence sums (Brown, 2002 ▸) using the parameters of Brese & O’Keeffe (1991 ▸) are much closer to the expected values of 4 for Sn and Te and 2 for O on basis of the current model [previous model: Sn1 4.28 valence units (v.u.), Te1 4.10 v.u., O1 2.09 v.u., O2 2.08 v.u.; current model: Sn1: 4.14 v.u., Te1 3.93 v.u., O1 1.99 v.u., O2 2.00 v.u.].

The relation of the isotypic crystal structures of *M* ^IV^Te_3_O_8_ compounds with that of the fluorite structure has been discussed previously for TiTe_3_O_8_ (Meunier & Galy, 1971 ▸; Wells, 1975 ▸). The unit-cell parameter *a* of cubic TiTe_3_O_8_ is ∼2*a* of cubic CaF_2_, whereby the ordered distribution of the cationic sites leads to a doubling of the unit cell and also to a considerable distortion of the respective coordination environments. The original cubic coordination around the Ca^II^ cation in the fluorite structure is changed to an octa­hedral coordination of Sn^IV^ and a fourfold coordination of Te^IV^ in the superstructure of the *M* ^IV^Te_3_O_8_ compounds. Note that there are two additional O atoms at a distance of 3.2446 (19) Å around the *M* ^IV^ site and two pairs of additional O atoms at a distance of 2.9076 (12) and 3.3957 (13) Å around the Te1 site in SnTe_3_O_8_, completing an eightfold coordination in each case. Correspondingly, each of the two O sites has a fourfold coordination in case the much longer distances are counted.

A qu­anti­tative structural comparison of the *M* ^IV^Te_3_O_8_ structures where single crystal data are available (*M* = Ti, Zr, Sn) was undertaken with the program *compstru* (de la Flor *et al.*, 2016 ▸) available at the Bilbao Crystallographic Server (Aroyo *et al.*, 2006 ▸). Table 2 ▸ lists the degree of lattice distortion (*S*), the maximum distance between the atomic positions of paired atoms (|*u*|), the arithmetic mean of all distances, and the measure of similarity (Δ) relative to SnTe_3_O_8_ as the reference structure. All these values show a very high similarity between the crystal structures in the isotypic *M* ^IV^Te_3_O_8_ series.

## Synthesis and crystallization

**Figure 3:**
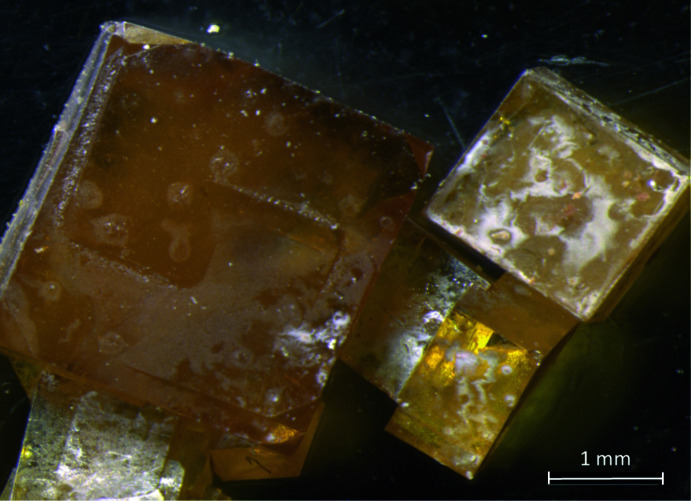
Photograph of Sn_3_TeO_8_ single crystals grown by chemical vapor transport reactions.

Reagent-grade chemicals were used without further purification. SnO_2_ (71 mg, 0.47 mmol) and TeO_2_ (225 mg, 1.40 mmol) were thoroughly mixed in the molar ratio 1:3 and placed in a silica tube to which 50 mg of TeCl_4_ were added as the transport agent. The silica ampoule was then evacuated and torch-sealed, placed in a two-zone furnace using a temperature gradient 973 K (source) → 873 K (sink) for three days. Cubic, canary-yellow crystals had formed in the millimetre size range in the colder sink region as the only product (Fig. 3 ▸). Powder X-ray diffraction of the remaining material in the source region revealed SnTe_3_O_8_ as the main phase and SnO_2_ as a side phase. For the single-crystal diffraction study, a fragment was broken from a larger crystal.

## Refinement

Crystal data, data collection and structure refinement details are summarized in Table 3 ▸. Atomic coordinates and the labelling scheme were adapted from isotypic TiTe_3_O_8_ (Meunier & Galy, 1971 ▸).

## Supplementary Material

Crystal structure: contains datablock(s) I. DOI: 10.1107/S2056989021011828/hb7998sup1.cif

Structure factors: contains datablock(s) I. DOI: 10.1107/S2056989021011828/hb7998Isup2.hkl

CCDC reference: 2120742

Additional supporting information:  crystallographic information; 3D view; checkCIF report

## Figures and Tables

**Table 1:** Selected geometric parameters (Å, °)

Sn1—O1^i^	2.0421 (11)	Te1—O2	2.1278 (3)
Te1—O1^ii^	1.8800 (11)		
			
O1^iii^—Te1—O1^ii^	102.42 (8)	O2^iv^—Te1—O2	157.05 (6)
O1^iii^—Te1—O2	86.60 (6)	Te1—O2—Te1^v^	117.94 (2)
O1^ii^—Te1—O2	79.05 (4)		

Symmetry codes: (i) -x+{\script{1\over 2}}, -y, z-{\script{1\over 2}}; (ii) -z+{\script{1\over 2}}, -x+{\script{1\over 2}}, -y+{\script{1\over 2}}; (iii) -z+{\script{1\over 2}}, x-{\script{1\over 2}}, y; (iv) x, -y, -z+{\script{1\over 2}}; (v) z, x, y.

**Table 2:** Atom pairs and their absolute distances |*u*| (Å) in the isotypic series *M*Te_3_O_8_ with SnTe_3_O_8_ as the reference structure, as well as degree of lattice distortion (*S*), arithmetic mean of the distances (*d*_av_, Å) and measure of similarity (Δ)

	TiTe_3_O_8_ *^*a*^*	(Ti_0.96_Fe_0.04_)Te_3_O_8_ *^*b*^*	ZrTe_3_O_8_ *^*c*^*	ZrTe_3_O_8_ *^*d*^*
*M* ^IV^1	0	0	0	0
Te1	0.0475	0.0360	0.0065	0.0059
O1	0.1061	0.0834	0.0713	0.0694
O2	0.1374	0.0968	0.0543	0.0446
*S*	0.0107	0.0102	0.0076	0.0092
*d*_av_	0.0878	0.0668	0.0453	0.0436
Δ	0.011	0.008	0.006	0.006

Notes: (*a*) *a* = 10.956 (3) Å; Meunier & Galy (1971 ▸); (*b*) *a* = 10.965 (1) Å; Bindi & Cipriani (2003 ▸); (*c*) *a* = 11.308 (1) Å; Noguera *et al.* (2003 ▸); (*d*) *a* = 11.340 (4) Å; Lu *et al.* (2019 ▸).

**Table 3:** Experimental details

Crystal data	
Chemical formula	SnTe_3_O_8_
*M*_r_	629.49
Crystal system, space group	Cubic, *I* *a*\overline{3}
Temperature (K)	296
*a* (Å)	11.1574 (4)
*V* (Å^3^)	1388.96 (15)
*Z*	8
Radiation type	Mo *K*α
μ (mm^−1^)	16.04
Crystal size (mm)	0.06 × 0.06 × 0.01

Data collection	
Diffractometer	Bruker APEXII CCD
Absorption correction	Multi-scan (*SADABS*; Krause *et al.*, 2015 ▸)
*T*_min_, *T*_max_	0.452, 0.748
No. of measured, independent and observed [*I* > 2σ(*I*)] reflections	14087, 735, 697
*R*_int_	0.048
(sin θ/λ)_max_ (Å^−1^)	0.907

Refinement	
*R*[*F* ^2^ > 2σ(*F* ^2^)], *wR*(*F* ^2^), *S*	0.014, 0.030, 1.07
No. of reflections	735
No. of parameters	21
Δρ_max_, Δρ_min_ (e Å^−3^)	1.27, −0.86

Computer programs: *APEX3* and *SAINT* (Bruker, 2018 ▸), *SHELXL* (Sheldrick, 2015 ▸), *ATOMS* (Dowty, 2006 ▸) and *publCIF* (Westrip, 2010 ▸).

## supplementary crystallographic information

### Crystal data

**Table:**

SnTe_3_O_8_	Mo *K*α radiation, λ = 0.71073 Å
*M**_r_* = 629.49	Cell parameters from 5266 reflections
Cubic, *I**a*3	θ = 3.7–38.9°
*a* = 11.1574 (4) Å	µ = 16.04 mm^−^^1^
*V* = 1388.96 (15) Å^3^	*T* = 296 K
*Z* = 8	Plate, light yellow
*F*(000) = 2160	0.06 × 0.06 × 0.01 mm
*D*_x_ = 6.021 Mg m^−^^3^	

### Data collection

**Table:**

Bruker APEXII CCD diffractometer	697 reflections with *I* > 2σ(*I*)
ω– and φ–scans	*R*_int_ = 0.048
Absorption correction: multi-scan (*SADABS*; Krause *et al.*, 2015)	θ_max_ = 40.2°, θ_min_ = 3.7°
*T*_min_ = 0.452, *T*_max_ = 0.748	*h* = −18→20
14087 measured reflections	*k* = −20→20
735 independent reflections	*l* = −19→20

### Refinement

**Table:**

Refinement on *F*^2^	0 restraints
Least-squares matrix: full	*w* = 1/[σ^2^(*F*_o_^2^) + (0.0127*P*)^2^ + 1.9293*P*] where *P* = (*F*_o_^2^ + 2*F*_c_^2^)/3
*R*[*F*^2^ > 2σ(*F*^2^)] = 0.014	(Δ/σ)_max_ = 0.001
*wR*(*F*^2^) = 0.030	Δρ_max_ = 1.27 e Å^−^^3^
*S* = 1.07	Δρ_min_ = −0.85 e Å^−^^3^
735 reflections	Extinction correction: SHELXL-2017/1 (Sheldrick 2015), Fc^*^=kFc[1+0.001xFc^2^λ^3^/sin(2θ)]^-1/4^
21 parameters	Extinction coefficient: 0.00046 (3)

### Special details

**Table:**

Geometry. All esds (except the esd in the dihedral angle between two l.s. planes) are estimated using the full covariance matrix. The cell esds are taken into account individually in the estimation of esds in distances, angles and torsion angles; correlations between esds in cell parameters are only used when they are defined by crystal symmetry. An approximate (isotropic) treatment of cell esds is used for estimating esds involving l.s. planes.

### Fractional atomic coordinates and isotropic or equivalent isotropic displacement parameters (Å^2^)

**Table:**

	*x*	*y*	*z*	*U*_iso_*/*U*_eq_	
Sn1	0.000000	0.000000	0.000000	0.00501 (4)	
Te1	0.20584 (2)	0.000000	0.250000	0.00804 (4)	
O1	0.43242 (10)	0.13738 (10)	0.39972 (11)	0.0129 (2)	
O2	0.16789 (10)	0.16789 (10)	0.16789 (10)	0.0078 (3)	

### Atomic displacement parameters (Å^2^)

**Table:**

	*U*^11^	*U*^22^	*U*^33^	*U*^12^	*U*^13^	*U*^23^
Sn1	0.00501 (4)	0.00501 (4)	0.00501 (4)	−0.00031 (3)	−0.00031 (3)	−0.00031 (3)
Te1	0.00518 (5)	0.01273 (6)	0.00620 (5)	0.000	0.000	−0.00229 (4)
O1	0.0095 (4)	0.0117 (4)	0.0174 (5)	0.0019 (3)	0.0018 (4)	0.0094 (4)
O2	0.0078 (3)	0.0078 (3)	0.0078 (3)	0.0020 (3)	0.0020 (3)	0.0020 (3)

### Geometric parameters (Å, º)

**Table:**

Sn1—O1^i^	2.0421 (11)	Sn1—O1^vi^	2.0421 (11)
Sn1—O1^ii^	2.0421 (11)	Te1—O1^v^	1.8800 (11)
Sn1—O1^iii^	2.0421 (11)	Te1—O1^vii^	1.8800 (11)
Sn1—O1^iv^	2.0421 (11)	Te1—O2^viii^	2.1278 (3)
Sn1—O1^v^	2.0421 (11)	Te1—O2	2.1278 (3)
			
O1^i^—Sn1—O1^ii^	86.09 (4)	O1^iv^—Sn1—O1^vi^	93.91 (4)
O1^i^—Sn1—O1^iii^	93.91 (4)	O1^v^—Sn1—O1^vi^	86.09 (4)
O1^ii^—Sn1—O1^iii^	180.00 (9)	O1^v^—Te1—O1^vii^	102.42 (8)
O1^i^—Sn1—O1^iv^	86.09 (4)	O1^v^—Te1—O2^viii^	79.05 (4)
O1^ii^—Sn1—O1^iv^	93.91 (4)	O1^vii^—Te1—O2^viii^	86.60 (6)
O1^iii^—Sn1—O1^iv^	86.09 (4)	O1^v^—Te1—O2	86.60 (6)
O1^i^—Sn1—O1^v^	93.91 (4)	O1^vii^—Te1—O2	79.05 (4)
O1^ii^—Sn1—O1^v^	86.09 (4)	O2^viii^—Te1—O2	157.05 (6)
O1^iii^—Sn1—O1^v^	93.91 (4)	Te1^ix^—O1—Sn1^x^	134.17 (6)
O1^iv^—Sn1—O1^v^	180.00 (9)	Te1—O2—Te1^xi^	117.94 (2)
O1^i^—Sn1—O1^vi^	180.00 (9)	Te1—O2—Te1^xii^	117.94 (2)
O1^ii^—Sn1—O1^vi^	93.91 (4)	Te1^xi^—O2—Te1^xii^	117.94 (2)
O1^iii^—Sn1—O1^vi^	86.09 (4)		

Symmetry codes: (i) *y*, −*z*+1/2, *x*−1/2; (ii) −*x*+1/2, −*y*, *z*−1/2; (iii) *x*−1/2, *y*, −*z*+1/2; (iv) *z*−1/2, −*x*+1/2, −*y*; (v) −*z*+1/2, *x*−1/2, *y*; (vi) −*y*, *z*−1/2, −*x*+1/2; (vii) −*z*+1/2, −*x*+1/2, −*y*+1/2; (viii) *x*, −*y*, −*z*+1/2; (ix) −*y*+1/2, −*z*+1/2, −*x*+1/2; (x) −*x*+1/2, −*y*, *z*+1/2; (xi) *z*, *x*, *y*; (xii) *y*, *z*, *x*.
